# Association of brain-derived neurotrophic factor levels at different trimesters and new-onset depressive symptom in the third trimester among pregnant women: a longitudinal study

**DOI:** 10.3389/fpsyt.2025.1618041

**Published:** 2025-07-31

**Authors:** Yue-rong Zhang, Ya-ping Liu, Xiang-mei Wu, Yuan Yan, Yan-feng Lou, Juan Ni

**Affiliations:** ^1^ Department of Respiratory and Critical Care, Day Surgery Management Center, Nanjing Jinling Hospital, Affiliated Hospital of Nanjing University, Nanjing, China; ^2^ Department of Obstetrics and Gynecology, Nanjing Jinling Hospital, Affiliated Hospital of Nanjing University, Nanjing, China; ^3^ Department of stomatology, Nanjing Jinling Hospital, Affiliated Hospital of Nanjing University, Nanjing, China

**Keywords:** pregnancy, pregnant women, depression, depressive symptom, BDNF

## Abstract

**Background:**

Accumulating evidence e suggests that brain-derived neurotrophic factor (BDNF) may play a role in the development of depression. However, changes in serum BDNF during distinct gestational periods and their association with prenatal depression remain unclear.

**Objectives:**

To investigate the change of serum BDNF in the first, second and third trimester and their longitudinal association with depressive symptoms in the third trimester.

**Methods:**

Depressive symptoms in the first and third trimester were assessed using the Patient Health Questionnaire-9 (PHQ-9). An automatic biochemical analyzer was used to detect serum BDNF levels based on enzyme-linked immunosorbent assay (ELISA) in the first, second and third trimester. Linear regression, binary and multivariable logistic regression model were used to analyze the association between BDNF levels during different pregnancy with PHQ-9 score and depressive symptoms in the third trimester.

**Results:**

The mean age of 500 pregnant women included in this study was (26.8 ± 2.3) years in the first trimester. At the third trimester, a total of 72 pregnant women (14.4%) developed depressive symptoms. The average serum BDNF level was highest in the first trimester and lowest in the second trimester. Each 1 pg/mL increase of first trimester BDNF was associated with a 43% decrease in the risk of prenatal depressive symptoms (95% confidence interval [95% *CI*]: 0.51, 0.65); each 1 pg/mL increase of second trimester BDNF was a 39% decrease in the risk of prenatal depressive symptoms (95% *CI*: 0.54, 0.68); each 1 pg/mL increase of third trimester BDNF was associated with a 36% decrease in the risk of prenatal depressive symptoms (95% *CI*: 0.58, 0.71).

**Conclusion:**

Overall, serum BDNF levels in the first, second, and third trimester were significantly associated with decreased PHQ-9 score and reduced risk of prenatal depressive symptoms. Serum BDNF shows promise as a predictive biomarker for antenatal depressive symptoms across all trimesters.

## 1 Introduction

Depression is one of the most common and disabling mental illnesses in the world, affecting more than 300 million people worldwide and often accompanied by severe functional impairment (1–3). Notably, depression among pregnant women is highly prevalent, and poses a substantial economic and public health burden (4–6). The prevalence of depression among pregnant women is approximately 20% (7), and 12% of these patients require clinical intervention or treatment (8). Accumulating evidence revealed that depression during pregnancy can have adverse effects on the pregnant woman and the fetus, including postpartum depression, gestational hypertension, spontaneous abortion, preterm birth, low birth weight, intrauterine growth restriction (5, 9–12). In extreme cases, maternal depression during pregnancy increases the risk of infant death due to neglect and abuse (13). Therefore, as one of the major public health issues, depression during pregnancy requires more research to identify its risk factors.

Brain derived neurotrophic factor (BDNF), as a member of the neurotrophic family, plays an important role in the development, maintenance, and functional regulation of the nervous system (14, 15). Importantly, BDNF is involved in a wide range of central processes, such as brain development, learning, memory or emotion regulation (16, 17). In recent years, the role of BDNF in the development of depression has aroused extensive attention (18–20). Evidence has suggested that BDNF may reduce the risk of depression through a range of mechanisms, including enhancing neuroplasticity, promoting neurogenesis, and modulating neurotransmitters (21, 22). Thus, additional population-based studies are necessary to explore the potential association between BDNF and depression.

Previous epidemiological studies mainly focused on the relationship between BDNF and postpartum depressive symptoms (within 3 months after delivery), indicating that the BDNF level of pregnant women with postpartum depressive symptoms is significantly lower than that of pregnant women without postpartum depressive symptoms, and the lower BDNF level is associated with an increased risk of postpartum depressive symptoms (23–26). However, to our knowledge, only one previous study has examined the association between BDNF in the first trimester and prenatal depressive symptoms, which showed that the risk of prenatal depressive symptom was 1.61-fold greater in the group with lower BDNF levels than in the group with higher BDNF levels (27). Although studies have investigated the association between BDNF levels in the first trimester and prenatal depressive symptoms, the pattern of changes in serum BDNF during different trimesters and its association with prenatal depressive symptoms is unclear. It is important to note that at different stages of pregnancy, physiological and hormonal levels change significantly, and these changes may directly or indirectly affect the expression and function of BDNF (28, 29). More research is therefore needed to investigate the dynamics of BDNF levels in different gestational periods and its association with prenatal depressive symptoms.

To address these gaps in knowledge, we performed a population-based longitudinal study among Chinese pregnant woman. The objective of the present study was to investigate the dynamic changes of serum BDNF levels in pregnant women during the first, second and third trimesters and its potential association with new-onset depressive symptoms in the third trimester.

## 2 Methods

### 2.1 Study design and participants

**Figure 1:**
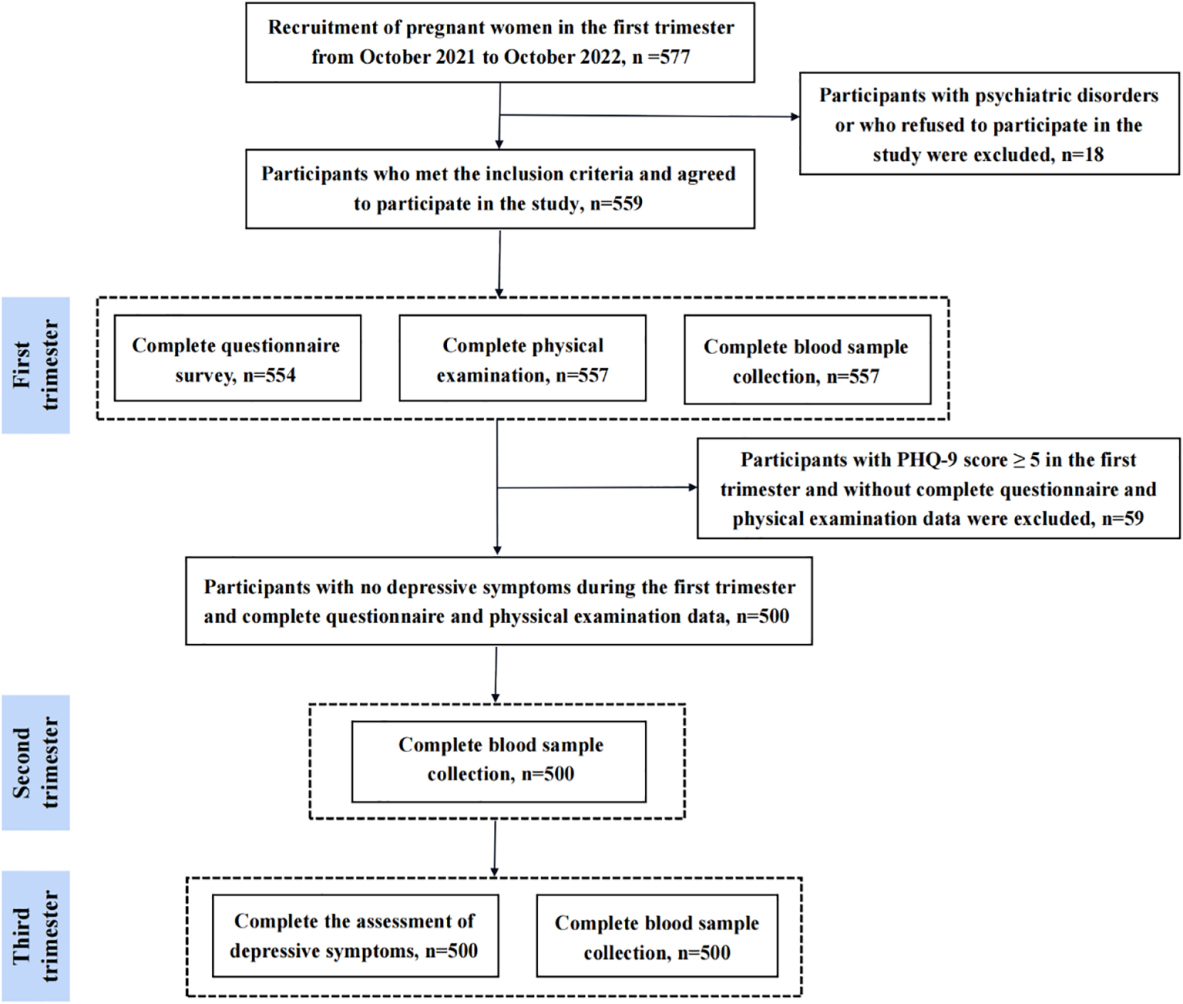
Study flow diagram.

Participants of the current study were enrolled from General Hospital of the eastern theater of the Chinese People’s Liberation Army (Nanjing, Jiangsu Province, China). In this study, pregnant women (within 12 weeks of pregnancy) who registered in our hospital and agreed to participate in this study were recruited in the obstetrics clinic by means of poster advertisements and introduction by outpatient physician. Inclusion criteria are as follows: i) pregnant women within 12 weeks of gestation; ii) registered at our hospital’s obstetrics clinic; iii) willing to participate and provide written informed consent; iv) no depressive symptoms at baseline. Exclusion criteria are as follows: i) any pre-existing psychiatric disorders (assessed by medical records or self-report); ii) voluntary withdrawal from the study; iii) incomplete questionnaire or physical examination data; iv) depressive symptoms at baseline. The flow diagram is shown in **Figure 1**. Briefly, a total of 577 Chinese early pregnant were recruited between October 2021 and October 2022. Of these, 18 participants were excluded due to having any psychiatric disorder, or voluntary withdrawal from the study. Among the 559 enrolled participants, 554 completed the questionnaire survey, 557 completed the physical examination and peripheral blood sample collection in the first trimester. Subsequently, 59 participants were excluded due to the following reasons: i) without complete questionnaire or physical examination data; ii) depressive symptoms at the first trimester. Consequently, 500 participants who had no depressive symptoms at the first trimester and complete questionnaire or physical examination data were included. At the second trimester, 500 participants completed the blood sample collection. At the third trimester, 500 participants completed the questionnaire survey and blood sample collection. Blood samples collected during the first, second, and third trimester were used to detect BDNF. Ultimately, a total of 500 participants with complete questionnaire data and blood samples were included in the analysis. This study was approved by ethics committee of Nanjing Jinling Hospital, Affiliated Hospital of Nanjing Medical University (Nanjing, China). All participants provided written informed consent.

### 2.2 Depressive symptoms assessment

The depressive symptoms of pregnant women in the first and third trimester were evaluated using the 9-item Patient Health Questionnaire (PHQ-9), a widely used screening instrument for depressive symptoms (30, 31). The total score of PHQ-9 ranges from 0 to 66, with higher scores indicating more severe depressive symptoms. The PHQ-9 score was used to determine whether the participants had depressive symptoms and the degree of depressive symptoms. Two cut-off values were set in this study: i) no depressive symptoms (PHQ-9 score ≤ 4), depressive symptoms (PHQ-9 score ≥ 5); ii) no depressive symptoms (0 ≤ PHQ-9 score ≤ 4), minor depressive symptoms (5 ≤ PHQ-9 score ≤ 9), mild depressive symptoms (10 ≤ PHQ-9 score ≤ 14), moderate-severe depressive symptoms (15 ≤ PHQ-9 score ≤ 27).

### 2.3 Peripheral serum BDNF measurement

Participants provided fasting blood samples (5 mL) of peripheral blood during the first, second and third trimester of pregnancy. The blood samples were centrifuged in a refrigerated centrifuge at 3000 rpm/min for 8 minutes to collect the serum. Serum was collected after centrifugation and stored in a −80°C freezer until assayed. The levels of serum BDNF in the first, second and third trimester were measured using an AU5400 automatic biochemical analyzer (Olympus Corporation, Japan). Serum BDNF levels were determined with enzyme-linked immunosorbent assay (ELISA) kits (DY248, RD Systems, USA) according to the manufacturer’s instructions.

### 2.4 Covariates

Covariates were selected based on prior knowledge and literature (18, 32). Information on covariates in the first trimester was obtained as following two ways: i) questionnaire, including age, marital status (married, unmarried, divorced), household economic conditions (poor, fair, good), educational level (high school and below, junior college, undergraduate and above), sleep duration, physical activity (< 1 h/d or ≥ 1 h/d), occupations (worker, clerk, teacher, farmer, medical professional, freelancer, other), and first trimester PHQ-9 score; ii) physical examination, body mass index (BMI) = weight (kg)/height (m) squared.

### 2.5 Statistical analysis

Sample characteristics were summarized as number (percentage) for categorical variables and as mean ± standard deviation (SD) for continuous variables. Statistical differences in sample characteristics between groups (non-depressive symptoms vs. depressive symptoms) were analyzed using independent-sample t-test for quantitative data and chi-squared test or Fisher exact probability test for categorical data. Differences in PHQ-9 score at the third trimester between different BDNF levels groups (Q1 vs. Q2, Q3, Q4) were tested by one-way analysis of variance (ANOVA). Correlations between BDNF in the first, second and third trimester with the PHQ-9 score in the first and third trimester were examined by the Pearson correlation analysis.

Univariable and multivariable linear regression analyses were conducted to assess the associations between BDNF levels in different trimesters and PHQ-9 score in the third trimester. In addition, binary and multivariable logistic regression models were used to evaluate the association between BDNF levels in different trimesters and depressive symptoms in the third trimester. The *β*, odds ratio (*OR*), and 95% confidence interval (*CI*) were reported. Three models were fitted in multivariable linear regression analyses and logistic regression analyses: model 1 was unadjusted; model 2 was adjusted for the first trimester age, marital status, household economic conditions, educational level, body mass index, sleep duration, physical activity, and occupations; model 3 was additionally adjusted for the first trimester PHQ-9 score. All analyses were completed using SPSS (version 27.0, Chicago, USA).

## 3 Results

### 3.1 Sample characteristics between-group differences

The characteristics of the study sample are shown in **Table 1**. The mean age of the 500 participants was 26.8 ± 2.3 years at the first trimester. At the third trimester follow-up survey, a total of 72 pregnant women (14.4%) developed depressive symptoms. Compared with the non-depressed group, individuals in the depressed group were younger, had higher rates of divorce and unmarried, slept longer, and had lower levels of physical activity (all *P*-values < 0.05). In addition, BDNF levels in the first, second and third trimester were significantly lower in the depressed group than in the non-depressed group (all *P*-values < 0.001).

**Table 1:** Sample characteristics.

Baseline characteristics	Total (N = 500)	New-onset depressive symptom at the third trimester		*P*-value
		**No (N = 428)**	**Yes (N = 72)**	
Age (years), mean ± SD	26.8 ± 2.3	27.0 ± 2.3	25.9 ± 2.3	< 0.001
Marital status, N (%)				0.024
Married	487 (97.4)	420 (98.1)	67 (93.1)	
Unmarried	7 (1.4)	5 (1.2)	2 (2.8)	
Divorced	6 (1.2)	3 (0.7)	3 (4.2)	
Household economic conditions, N (%)				0.540
Poor	134 (26.8)	113 (26.4)	21 (29.2)	
Fair	342 (68.4)	296 (69.2)	46 (63.9)	
Good	24 (4.8)	19 (4.4)	5 (7.0)	
Educational level, N (%)				0.501
High school and below	243 (48.6)	204 (47.7)	39 (54.2)	
Junior college	82 (16.4)	73 (17.1)	9 (12.5)	
Undergraduate and above	175 (35.0)	151 (35.3)	24 (33.3)	
Body mass index (kg/m^2^), mean ± SD	22.1 ± 3.3	22.0 ± 3.2	22.3 ± 3.8	0.495
Sleep duration (h/d), mean ± SD	7.6 ± 0.9	7.6 ± 0.8	7.9 ± 1.0	0.006
Physical activity, N (%)				0.039
< 1 h/d	391 (78.2)	328 (76.6)	63 (87.5)	
≥ 1 h/d	109 (21.8)	100 (23.4)	9 (12.5)	
Occupations, N (%)				0.374
Worker	80 (16.0)	66 (15.4)	14 (19.5)	
Clerk	75 (15.0)	69 (16.1)	6 (8.3)	
Teacher	66 (13.2)	59 (13.8)	7 (9.7)	
Farmer	22 (4.4)	17 (4.0)	5 (6.9)	
Medical professional	85 (17.0)	73 (17.1)	12 (16.7)	
Freelancer	137 (27.4)	113 (26.4)	24 (33.3)	
Other	35 (7.0)	31 (7.2)	4 (5.6)	
First trimester BDNF (pg/mL), mean ± SD	11.3 ± 3.5	12.0 ± 3.0	6.7 ± 2.9	< 0.001
Second trimester BDNF (pg/mL), mean ± SD	10.6 ± 3.8	11.2 ± 3.4	6.6 ± 3.4	< 0.001
Third trimester BDNF (pg/mL), mean ± SD	11.0 ± 4.1	11.9 ± 3.6	6.1 ± 3.5	< 0.001

SD, standard deviation; BDNF, brain-derived neurotrophic factor; Bold font refers to statistically significant *P*-values; Depressive symptoms were evaluated using the PHQ-9 scale, with a total score range of 0–27 points, and the higher the total score, the more severe the depressive symptoms. PHQ-9 score ≥ 5 represented suffering depressive symptoms.

**Figure 2:**
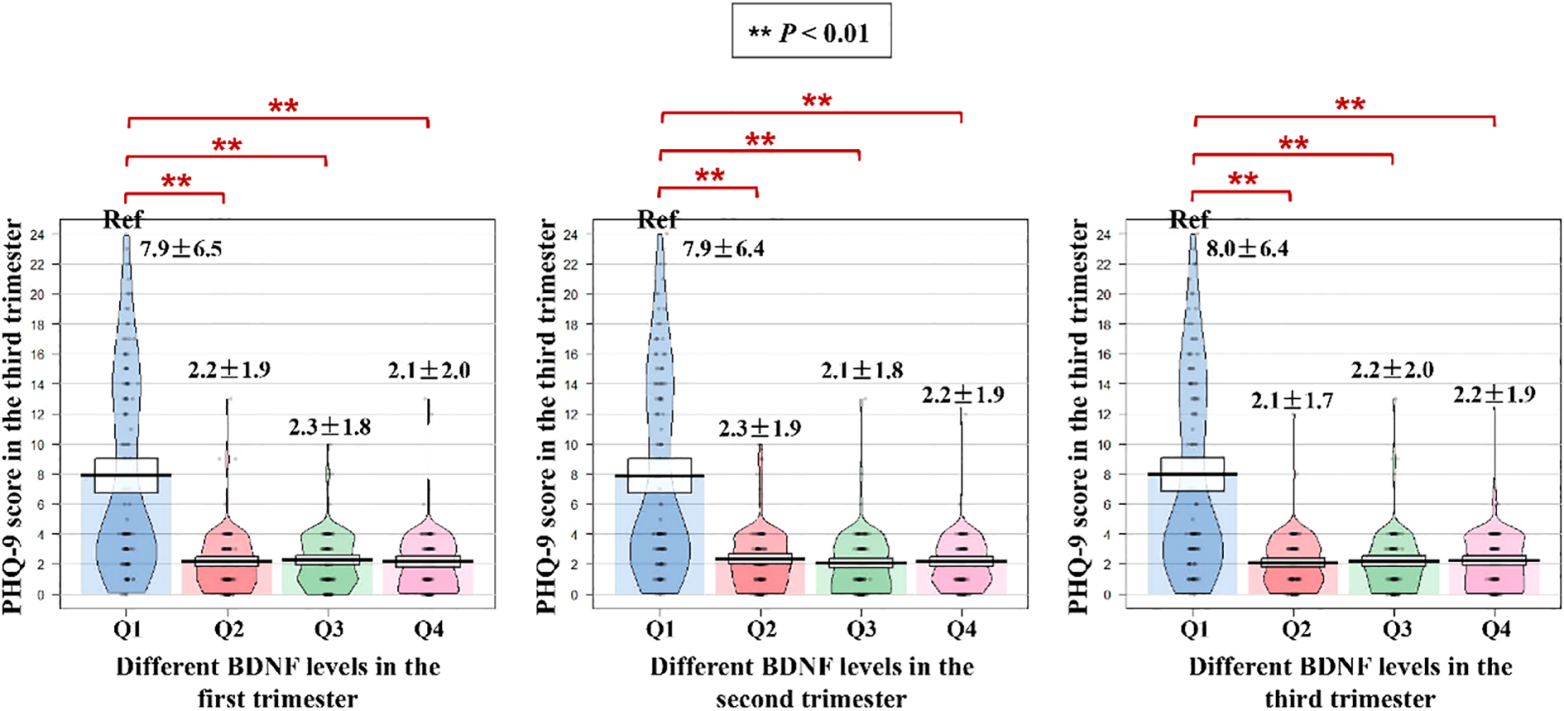
Stratified distribution of PHQ-9 scores in the third trimester by BDNF level quartiles across pregnancy trimesters. PHQ-9, Patient Health Questionnaire-9; BDNF, brain-derived neurotrophic factor. ** P<0.01.

The distribution of PHQ-9 score in the third trimester among different BDNF levels groups is shown in **Figure 2**. Compared with the BDNF Q1 group in the first trimester, the PHQ-9 score in the third trimester was significantly lower in the Q2, Q3 and Q4 groups (Q1 vs. Q2 vs. Q3 vs. Q4 = 7.9 ± 6.5 points vs. 2.2 ± 1.9 points vs. 2.3 ± 1.8 points vs. 2.1 ± 2.0 points; all *P*-values < 0.01). Similarly, compared with the BDNF Q1 group in the second and third trimesters, the PHQ-9 score in the third trimester was significantly lower in the Q2, Q3 and Q4 groups (all *P*-values < 0.01).

### 3.2 Pearson correlation analysis and linear regression model

The results of correlation analysis are presented in **Supplementary Table S1**. Serum BDNF levels in the first, second and third trimesters were negatively correlated with PHQ-9 score in the first and third trimesters (correlation coefficients between −0.54 and −0.09; all *P*-values < 0.05).

**Figure 3:**
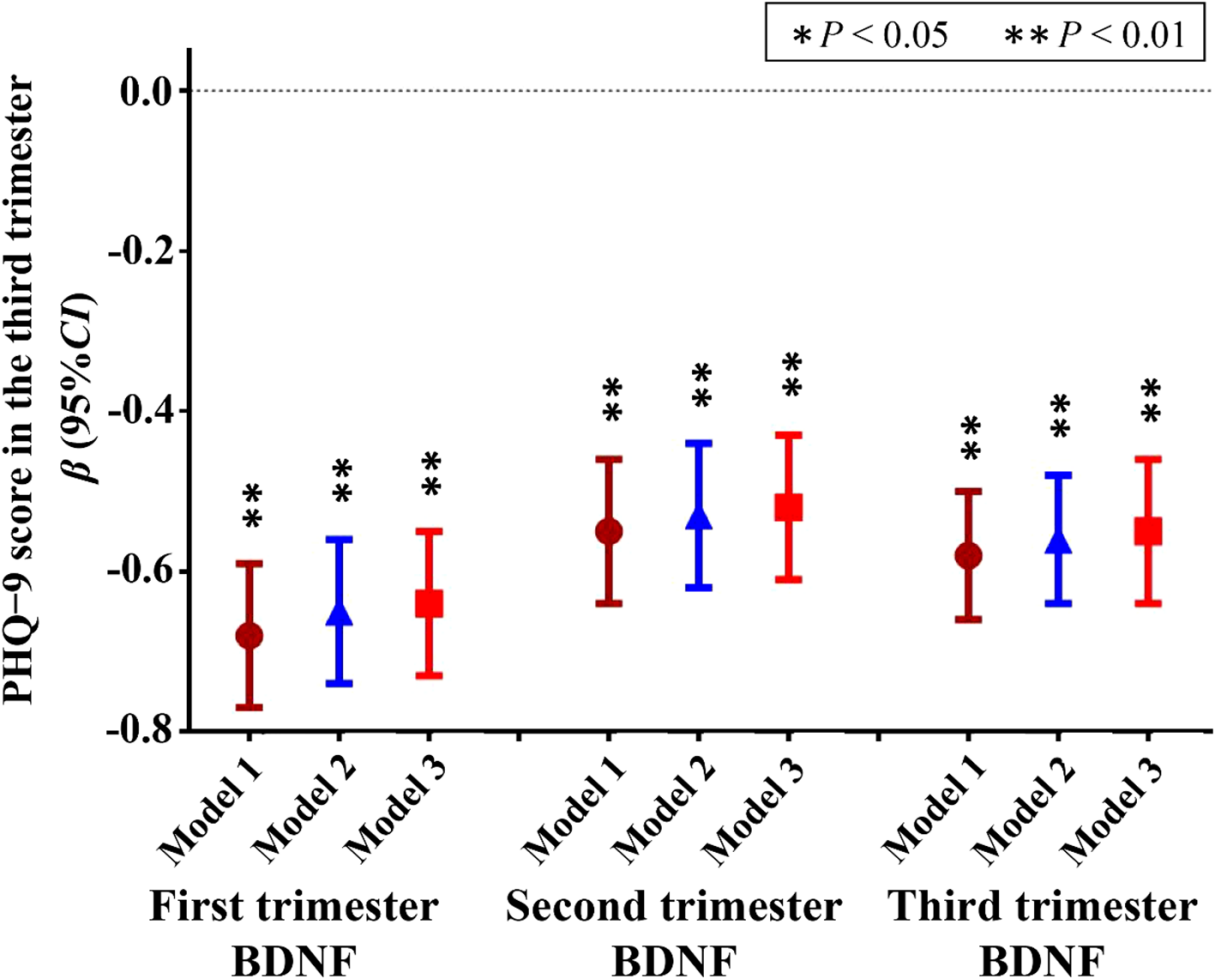
Linear regression model between BDNF levels during pregnancy and PHQ-9 score in the third trimester. BDNF, brain-derived neurotrophic factor; PHQ-9, Patient Health Questionnaire-9; Depressive symptoms were evaluated using the PHQ-9 scale, with a total score range of 0–27 points, and the higher the total score, the more severe the depressive symptoms; Model 1, unadjusted model; Model 2 Adjusted for first trimester age, marital status, household economic conditions, educational level, body mass index, sleep duration, physical activity, and occupations; Model 3, Model 2 further adjusted for the first trimester PHQ-9 score. * P<0.05, ** P<0.01.

The results of linear regression analysis are depicted in **Figure 3**; **Supplementary Table S2**. In multivariable linear regression models, after adjusting for the first trimester age, marital status, household economic conditions, educational level, body mass index, sleep duration, physical activity, occupations, and the first trimester PHQ-9 score, each 1 pg/mL increase of first trimester BDNF was associated with 0.64 point-decrease in PHQ-9 score in the third trimester (*β* = −0.64; 95% *CI*: −0.73, −0.55; *P* < 0.001); Each 1 pg/mL increase of second trimester BDNF was associated with 0.52 point-decrease in PHQ-9 score in the third trimester (*β* = −0.52; 95% *CI*: −0.61, −0.43; *P* < 0.001); Each 1 pg/mL increase of third trimester BDNF was associated with 0.55 point-decrease in PHQ-9 score in the third trimester (*β* = −0.55; 95% *CI*: −0.64, −0.46; *P* < 0.001). Results from unadjusted and adjusted models were generally consistent (**Figure 3**; **Supplementary Table S2**).

### 3.3 Binary and multivariable logistic regression models

**Table 2** demonstrates the results of binary logistic regression analysis. After adjusting for covariates, each 1 pg/mL increase of first trimester BDNF was associated with a 43% decrease in the risk of depressive symptoms in the third trimester (*OR* = 0.57, 95% *CI*: 0.51, 0.65); each 1 pg/mL increase of second trimester BDNF was a 39% decrease in the risk of depressive symptoms in the third trimester (*OR* = 0.61, 95% *CI*: 0.54, 0.68); each 1 pg/mL increase of third trimester BDNF was associated with a 36% decrease in the risk of depressive symptoms in the third trimester (*OR* = 0.64, 95% *CI*: 0.58, 0.71).

**Table 2:** Binary logistic regression analysis between BDNF levels during pregnancy and depressive symptoms in the third trimester.

BDNF levels during pregnancy	Model 1 ^a^		Model 2 ^b^		Model 3 ^c^	
	*OR* (95%*CI*)	*P*-value	*OR* (95%*CI*)	*P*-value	*OR* (95%*CI*)	*P*-value
First trimester BDNF	0.59 (0.53, 0.66)	<0.001	0.58 (0.52, 0.65)	<0.001	0.57 (0.51, 0.65)	<0.001
Second trimester BDNF	0.63 (0.57, 0.69)	<0.001	0.61 (0.55, 0.68)	<0.001	0.61 (0.54, 0.68)	<0.001
Third trimester BDNF	0.67 (0.61, 0.73)	<0.001	0.65 (0.59, 0.72)	<0.001	0.64 (0.58, 0.71)	<0.001

BDNF, brain-derived neurotrophic factor. Depressive symptoms were evaluated using the PHQ-9 scale, with a total score range of 0–27 points, and the higher the total score, the more severe the depressive symptoms. PHQ-9 score ≥ 5 was defined as depressive symptoms, PHQ-9 score ≤ 4 was considered as no depressive symptoms.

a Model 1, unadjusted model.

b Model 2 adjusted for first trimester age, marital status, household economic conditions, educational level, body mass index, sleep duration, physical activity, and occupations.

c Model 3, model 2 further adjusted for the first trimester PHQ-9 score.

In adjusted multivariable logistic regression models (**Table 3**), each 1 pg/mL increase of first trimester BDNF was associated with 32% lower risk for minor depressive symptoms in the third trimester (*OR* = 0.68, 95% *CI*: 0.59, 0.79), 46% lower risk for mild depressive symptoms in the third trimester (*OR* = 0.54, 95% *CI*: 0.45, 0.65), 59% lower risk for moderate-severe depressive symptoms in the third trimester (*OR* = 0.41, 95% *CI*: 0.30, 0.56); each 1 pg/mL increase of second trimester BDNF was associated with 24% lower risk for minor depressive symptoms in the third trimester (*OR* = 0.76, 95% *CI*: 0.65, 0.90), 40% lower risk for mild depressive symptoms in the third trimester (*OR* = 0.60, 95% *CI*: 0.50, 0.72), 76% lower risk for moderate-severe depressive symptoms in the third trimester (*OR* = 0.24, 95% *CI*: 0.14, 0.42); each 1 pg/mL increase of third trimester BDNF was associated with 20% lower risk for minor depressive symptoms in the third trimester (*OR* = 0.80, 95% *CI*: 0.70, 0.92), 42% lower risk for mild depressive symptoms in the third trimester (*OR* = 0.58, 95% *CI*: 0.49, 0.70), 85% lower risk for moderate-severe depressive symptoms in the third trimester (*OR* = 0.15, 95% *CI*: 0.07, 0.33).

**Table 3:** Multivariable logistic regression analysis between BDNF levels during pregnancy and depressive symptoms in the third trimester.

Variables	Model 1^a^		Model 2^b^		Model 3^c^	
	*OR* (95%*CI*)	*P*-value	*OR* (95%*CI*)	*P*-value	*OR* (95%*CI*)	*P*-value
First trimester BDNF						
No depressive symptoms	Ref		Ref		Ref	
Minor depressive symptoms	0.69 (0.60, 0.80)	<0.001	0.68 (0.59, 0.79)	<0.001	0.68 (0.59, 0.79)	<0.001
Mild depressive symptoms	0.57 (0.49, 0.68)	<0.001	0.55 (0.46, 0.66)	<0.001	0.54 (0.45, 0.65)	<0.001
Moderate-severe depressive symptoms	0.46 (0.35, 0.60)	<0.001	0.42 (0.31, 0.57)	<0.001	0.41 (0.30, 0.56)	<0.001
Second trimester BDNF						
No depressive symptoms	Ref		Ref		Ref	
Minor depressive symptoms	0.78 (0.67, 0.90)	0.001	0.76 (0.65, 0.89)	0.001	0.76 (0.65, 0.90)	0.001
Mild depressive symptoms	0.65 (0.56, 0.75)	<0.001	0.61 (0.52, 0.72)	<0.001	0.60 (0.50, 0.72)	<0.001
Moderate-severe depressive symptoms	0.34 (0.24, 0.49)	<0.001	0.28 (0.18, 0.45)	<0.001	0.24 (0.14, 0.42)	<0.001
Third trimester BDNF						
No depressive symptoms	Ref		Ref		Ref	
Minor depressive symptoms	0.82 (0.73, 0.92)	0.001	0.81 (0.71, 0.92)	0.001	0.80 (0.70, 0.92)	0.001
Mild depressive symptoms	0.65 (0.57, 0.74)	<0.001	0.61 (0.52, 0.71)	<0.001	0.58 (0.49, 0.70)	<0.001
Moderate-severe depressive symptoms	0.29 (0.18, 0.47)	<0.001	0.19 (0.09, 0.38)	<0.001	0.15 (0.07, 0.33)	<0.001

BDNF, brain-derived neurotrophic factor. Depressive symptoms were evaluated using the PHQ-9 scale, with a total score range of 0–27 points, and the higher the total score, the more severe the depressive symptoms. The PHQ-9 scores of 0 to 4, 5to 9, 10 to 14, 15 to 27 were defined as no depressive symptoms, minor, mild, moderate-severe depressive symptoms, respectively.

a Model 1, unadjusted model.

b Model 2 adjusted for first trimester age, marital status, household economic conditions, educational level, body mass index, sleep duration, physical activity, and occupations.

c Model 3, model 2 further adjusted for the first trimester PHQ-9 score.

## 4 Discussion

In this population-based longitudinal study, the average serum BDNF level was higher in the first trimester and lower in the second trimester. In addition, serum BDNF levels in the first, second, and third trimester were significantly associated with decreased PHQ-9 score and reduced risk of depressive symptom in the third trimester. Specifically, each 1 pg/mL increase in serum BDNF levels in the first, second, and third trimester was associated with 0.64, 0.52 and 0.55 point-decrease in PHQ-9 score in the third trimester; each 1 pg/mL increase in serum BDNF levels in the first, second, and third trimester was associated with a 43%, 39%, and 36% lower risk of depressive symptoms in the third trimester.

The results of this study suggest that BDNF in the first, second and third trimesters of pregnancy is a risk factor for prenatal depressive symptoms. This predictive value persists after adjusting for key covariates, suggesting BDNF’s potential as a clinical screening tool. As far as we know, only one previous study has explored the association between BDNF levels and prenatal depressive symptoms among pregnant women. Fung et al. (27) established that depressed pregnant women had significantly lower early-gestation BDNF levels than controls. Moreover, compared to the group with higher early-gestation BDNF levels (>25.31 ng/mL), the risk of prenatal depressive symptoms in the group with lower BDNF levels (≤ 25.31 ng/mL) increased by 61%. Our results align with but extend prior work by Fung et al. (27) who only examined the association between first-trimester BDNF and depressive symptoms. However, no study has investigated the association between BDNF in serum during distinct gestational periods and prenatal depressive symptoms. Consequently, more studies are needed to investigate the association between BDNF levels in different gestational periods with prenatal depressive symptoms, and the potential mechanisms.

Our longitudinal data address a critical knowledge gap by characterizing trimester-specific BDNF dynamics during pregnancy. The observed trimester-specific BDNF fluctuations (higher in the first trimester of pregnancy and lower in the second trimester) suggests complex regulation by gestational physiology. These findings underscore the need for standardized sampling protocols and reference ranges to better understand BDNF’s role in perinatal mental health. The placenta serves as a significant extra-neural source of BDNF during pregnancy (33), with secretion patterns potentially modulated by gestational hormones including estrogen and progesterone (34). This placental contribution may explain the characteristic U-shaped trajectory observed in our cohort. Additionally, reduced physical activity during pregnancy represents another potential modulator of BDNF levels (35, 36). While our study did not directly measure exercise parameters, the mid-pregnancy BDNF nadir corresponds with typical reductions in maternal activity during the second trimester. Future studies should incorporate objective activity monitoring and high-frequency sampling approaches to elucidate the mechanisms underlying these fluctuations.

The association between serum BDNF levels during distinct gestational periods and prenatal depression is biologically plausible, and several mechanisms may be involved. On the one hand, BDNF promotes the growth, development, and survival of neurons, increases the number of neurons and synaptic connections, thereby enhancing neuroplasticity (37, 38). Reduced neuroplasticity has been shown to be associated with an increased risk of depression (39, 40). On the other hand, BDNF can induce the proliferation and differentiation of neural stem cells and promote neurogenesis (41, 42). Previous studies have shown that patients with depression have hippocampal atrophy (e.g., decreased neurogenesis), and reduced BDNF levels may be one of the reasons for the decreased neurogenesis (43, 44). Additionally, BDNF can affect neurotransmitter function by regulating neurotransmitter synthesis, release, and reuptake (22). The monoamine hypothesis suggests that depression is caused by an imbalance of neurotransmitters such as norepinephrine and dopamine, and that reduced levels of BDNF may be one of the causes of the neurotransmitter imbalance (45, 46). Our trimester-specific findings suggest these protective mechanisms operate throughout pregnancy. The specific mechanism behind the association between BDNF levels and prenatal depression need further investigations.

Some limitations of this study deserve mention. Firstly, the observational nature of the current study precludes causal inference. Secondly, the sample source of this study was single (the analysis included only pregnant women), and the results cannot be generalized to the general population. Future multi-center studies with larger sample size are needed. Thirdly, in the present study, depressive symptoms were assessed subjectively by the PHQ-9 scale. Although the PHQ-9 scale has been widely used and proven to be an effective screening tool for depressive symptoms in adults, it can still cause misclassification of depressive symptoms (47, 48). Finally, even though we adjusted for many potential confounders in linear regression and logistic regression models, residual confounding could not be ruled out. In addition, the retention rate of the sample in this study is relatively high. Although it can enhance internal validity, the motivation level of the participants was not included as a confounding factor. Therefore, additional studies of population-wide cohorts are needed to validate our findings.

## 5 Conclusion

In summary, our results show that serum BDNF levels in the first, second, and third trimester were significantly associated with decreased PHQ-9 score and reduced risk of depressive symptoms in the third trimester. These consistent findings suggest BDNF may serve as both a potential biomarker and possible intervention target in perinatal mental health, though further research is needed to explore its clinical translation.

## Data Availability

The raw data supporting the conclusions of this article will be made available by the authors, without undue reservation.
