# Supplementary material for: Association of brain-derived neurotrophic factor levels at different trimesters and new-onset depressive symptom in the third trimester among pregnant women: a longitudinal study
Source: Front Psychiatry. 2025 Jul 31;16:1618041. doi: 10.3389/fpsyt.2025.1618041 (PMC12351285; doi:10.3389/fpsyt.2025.1618041)
Supplement: Supplementary file 1 [file Table1.docx]

**Table S1.** Correlations between BDNF levels during pregnancy with PHQ-9 score in the first and third trimester

| **Variables** | **First trimester BDNF** | **Second trimester BDNF** | **Third trimester BDNF** | **First trimester PHQ-9 score** | **Third trimester PHQ-9 score** |
| --- | --- | --- | --- | --- | --- |
| **First trimester BDNF** | 1 |  |  |  |  |
| **Second trimester BDNF** | 0.57^**^ | 1 |  |  |  |
| **Third trimester BDNF** | 0.69^**^ | 0.58^**^ | 1 |  |  |
| **First trimester PHQ-9 score** | -0.13^**^ | -0.09^*^ | -0.13^**^ | 1 |  |
| **Third trimester PHQ-9 score** | -0.55^**^ | -0.477^**^ | -0.54^**^ | 0.16^**^ | 1 |

Note: BDNF, brain-derived neurotrophic factor; Depressive symptoms were evaluated using the PHQ-9 scale, with a total score range of 0-27 points, and the higher the total score, the more severe the depressive symptoms; ^*^ *P*-value < 0.05, ^**^ *P*-value < 0.01.

**Table S2.** Linear regression model between BDNF levels during pregnancy and PHQ-9 score in the third trimester

| **BDNF levels during pregnancy** | **Model 1 ^a^** | |  | **Model 2 ^b^** | |  | **Model 3 ^c^** | |
| --- | --- | --- | --- | --- | --- | --- | --- | --- |
|  | ***β* (95%*CI*)** | ***P*-value** |  | ***β* (95%*CI*)** | ***P*-value** |  | ***β* (95%*CI*)** | ***P*-value** |
| **First trimester BDNF** | -0.68 (-0.77, -0.59) | <0.001 |  | -0.65 (-0.74, -0.56) | <0.001 |  | -0.64 (-0.73, -0.55) | <0.001 |
| **Second trimester BDNF** | -0.55 (-0.64, -0.46) | <0.001 |  | -0.53 (-0.62, -0.44) | <0.001 |  | -0.52 (-0.61, -0.43) | <0.001 |
| **Third trimester BDNF** | -0.58 (-0.66, -0.50) | <0.001 |  | -0.56 (-0.64, -0.48) | <0.001 |  | -0.55 (-0.64, -0.46) | <0.001 |

Note: BDNF, brain-derived neurotrophic factor. Depressive symptoms were evaluated using the PHQ-9 scale, with a total score range of 0-27 points, and the higher the total score, the more severe the depressive symptoms.

^a^ Model 1, unadjusted model.

^b^ Model 2 adjusted for first trimester age, marital status, household economic conditions, educational level, body mass index, sleep duration, physical activity, and occupations.

^c^ Model 3, model 2 further adjusted for the first trimester PHQ-9 score.
